# A Flexible Fringe Projection Vision System with Extended Mathematical Model for Accurate Three-Dimensional Measurement

**DOI:** 10.3390/s16050612

**Published:** 2016-04-28

**Authors:** Suzhi Xiao, Wei Tao, Hui Zhao

**Affiliations:** School of Electronic Information and Electrical Engineering, Shanghai Jiao Tong University, 800 Dongchuan Road Minhang District, Shanghai 200240, China; vsxiao@sjtu.edu.cn (S.X.); huizhao@sjtu.edu.cn (H.Z.)

**Keywords:** three-dimensional (3D) measurement, fringe projection, mathematical model extension, phase to 3D coordinates, least-squares parameter estimation, phase-coding

## Abstract

In order to acquire an accurate three-dimensional (3D) measurement, the traditional fringe projection technique applies complex and laborious procedures to compensate for the errors that exist in the vision system. However, the error sources in the vision system are very complex, such as lens distortion, lens defocus, and fringe pattern nonsinusoidality. Some errors cannot even be explained or rendered with clear expressions and are difficult to compensate directly as a result. In this paper, an approach is proposed that avoids the complex and laborious compensation procedure for error sources but still promises accurate 3D measurement. It is realized by the mathematical model extension technique. The parameters of the extended mathematical model for the ’phase to 3D coordinates transformation’ are derived using the least-squares parameter estimation algorithm. In addition, a phase-coding method based on a frequency analysis is proposed for the absolute phase map retrieval to spatially isolated objects. The results demonstrate the validity and the accuracy of the proposed flexible fringe projection vision system on spatially continuous and discontinuous objects for 3D measurement.

## 1. Introduction

Industrial safety inspection has recently received considerable attention in the academic community and technology companies. The accurate three-dimensional (3D) measurement provides important information on monitoring workplaces for industrial safety. Numerous techniques on 3D measurement have been studied, including Stereo-vision, Time-of-Flight, and structured light technique. Among these techniques, the phase-shifting digital fringe projection is widely recognized and studied due to its high resolution, high speed, and noncontact property [1,2]. In a fringe projection vision system, the projector projects light patterns with a sinusoidally changing intensity onto the detected objects, and the pattern images are deformed because of the objects${}^{\prime }$ physical profile; then, a camera captures the deformed pattern images. The absolute phase map on the detected objects is derived by the phase unwrapping of the captured deformed fringe patterns. The 3D information is uniquely extracted on the basis of the mapping relationship between the image coordinates and the corresponding absolute phase value. Considering the digital projector has a large working range, the fringe projection technique can be used on large scene reconstruction for industrial safety inspection.

In contrast to the polynomial fitting method [3,4] and least-squares method [5,6,7], the phase-to-depth transformation based on the geometrical modeling method [8,9,10,11,12,13] has been widely studied and used in the phase-shifting structured light technique owing to several advantages. First, no additional gauge block or precise linear z stage is needed for calibration, which simplifies the calibration process and offers more flexibility and a cost savings. Second, there can be no restriction on the camera’s and projector’s relative alignment regarding parallelism and perpendicularity. Third, it has a clear and explanatory mathematical expression for the phase to 3D coordinates transformation since it is derived from the triangulation relationship of the camera, projector, and detected object.

Although it has the advantages mentioned above, the given mathematical expression does not have sufficient accuracy because the adopted simplified geometrical model ignores the lens distortion and lens defocus of the fringe projection system. On the other hand, there are some other error sources that exist in the vision system that influence the accuracy of the 3D measurement. As a result, a large amount of work has been devoted to improving the measurement accuracy by considering the lens distortion [5,14], the gamma nonlinearity for phase error elimination [15,16], the high-order harmonics for phase extraction [17], the ambient light for phase error compensation [18], *etc*.

The extended mathematical model proposed in this paper eliminates the uncertainty in the calibration parameters and the uncertainty in the phase extraction. In addition, it will not incur any computational burden during normal computation of the phase to depth transformation. It avoids the time-consuming iterative operation and exhibits more precision.

Experiments are carried out for a single object and multiple spatially discontinuous objects with the proposed flexible fringe projection vision system by the extended mathematical model. For spatially isolated objects, the fringe orders will be ambiguous for the phase unwrapping, which makes it difficult to retrieve the absolute phase directly for three-step phase-shifting technique. As a result, the depth difference between spatially isolated surfaces is indiscernible. Therefore, in order to obtain an accurate 3D measurement of multiple discontinuous objects, several approaches are developed to derive an accurate absolute phase map. The gray-coding plus phase-shifting technique is one of the better methods to obtain the fringe order [19]. However, at least several more patterns are required to generate the appropriate number of codewords, which slows down the computational timing. In addition, Su [20] generated sinusoidal fringe patterns by giving every period a certain color, and the fringe order was provided by the color information of the fringe patterns. However, the phase accuracy might be influenced by the poor color response and color crosstalk. Wang *et al.* [21] presented a phase-coding method. Instead of encoding the codeword into binary intensity images, the codeword is embedded into the phase range of phase-shifted fringe images. This technique has the advantages of less sensitivity to the surface contrast, ambient light, and camera noise. However, three more frames are necessary for the phase-coding method. In this research, in order to realize the accurate 3D measurement of multiple discontinuous objects with the abovementioned extended mathematical model, we propose a new phase-coding method to obtain the absolute phase map by only adding one additional fringe pattern.

The structure of this paper is as follows. Section 2 reviews the principles for obtaining the 3D coordinates from the absolute phase. Section 3 introduces the mathematical model extension with least-squares parameter estimation. Section 4 presents the realization of the flexible fringe projection vision system for the accurate 3D measurement of a single continuous object by the extended mathematical model. Section 5 introduces the new phase-coding method for deriving an accurate absolute phase map for spatially isolated objects. Finally, the conclusions and the plans for future work are summarized.

## 2. Principle on Absolute Phase to 3D Coordinates Transformation

**Figure 1:**
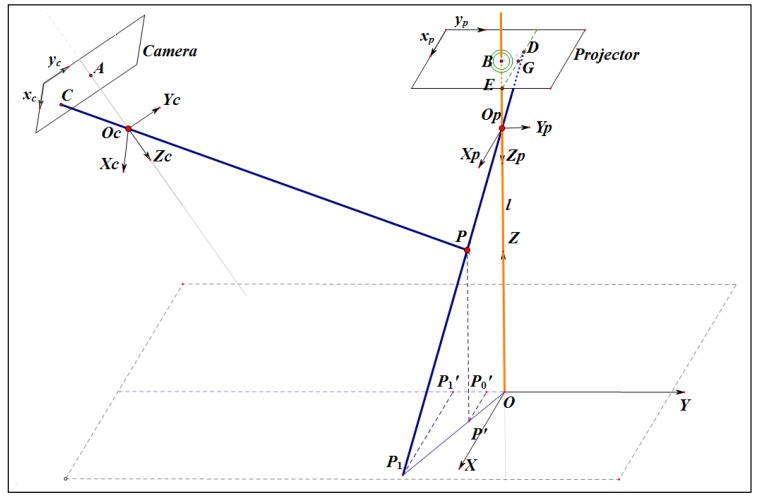
Geometrical model for the phase to depth transformation.

The geometrical model of the proposed fringe projection measurement system is shown in Figure 1. The camera and the projector are all described by a pinhole model. The camera imaging plane and the projection plane are arbitrarily arranged. The reference plane OXY is an imaginary plane, which is parallel to the projection plane. The projector coordinate system is denoted as $O_{p}X_{p}Y_{p}Z_{p}$. The camera coordinate system is denoted as $O_{c}X_{c}Y_{c}Z_{c}$. The imagined reference plane coordinate system is denoted as OXYZ denotes. *P* represents an arbitrary point on the detected object. (x,y,z), $\left(x^{c},y^{c},z^{c}\right)$, $\left(x^{p},y^{p},z^{p}\right)$ denote the imaginary reference coordinate, the camera coordinate and the projector coordinate of the point *P*, respectively. The imaging point of the point *P* is indicated as the point *C*. The point *D* indicates the fringe point which projects at the point *P* in space. The points A and B denote the lens center of the camera and the projector, respectively. Line $\bar{ED}$ is one of the sinusoidal fringes which is parallel to $x_{p}$ - axis. Line $\bar{BG}$ is vertical to Line $\bar{ED}$. Line $\bar{PP^{{}^{\prime }}}$ is parallel to *Z*-axis and crosses the imaginary reference plane at point $P^{{}^{\prime }}$. $\bar{PP_{1}}$ is the extension line of ray $\bar{DP}$ and crosses the imaginary reference plane at point $P_{1}$ . $\bar{P^{{}^{\prime }}P_{0}^{{}^{\prime }}}$ and $\bar{P_{1}P_{1}^{{}^{\prime }}}$ are parallel to *X*-axis and cross the *Y*-axis at point $P_{0}^{{}^{\prime }}$ and $P_{1}^{{}^{\prime }}$, respectively. The mathematical description of the phase to 3D coordinates transformation [22] is as follows:

(1) $$z^{c}=\frac{K\left(r_{21}^{-1}t_{x}+r_{22}^{-1}t_{y}+r_{23}^{-1}t_{z}\right)-\left(r_{31}^{-1}t_{x}+r_{32}^{-1}t_{y}+r_{33}^{-1}t_{z}\right)}{K\left(r_{21}^{-1}D_{1}+r_{22}^{-1}D_{2}+r_{23}^{-1}\right)-\left(D_{1}r_{31}^{-1}+D_{2}r_{32}^{-1}+r_{33}^{-1}\right)}\;$$

where $K=\frac{2\pi f^{p}}{\left(\varphi -\varphi_{0}\right)\lambda_{0}}\;$, $D_{1}=\frac{u_{c}-u_{0}^{c}}{f_{x}^{c}}\;$ , and $D_{2}=\frac{v_{c}-v_{0}^{c}}{f_{y}^{c}}\;$. Furthermore, the coordinates $\left(x^{c},y^{c}\right)$ at the given pixel $\left(u_{c},v_{c}\right)$ are obtained by the calculated depth $z^{c}$ as follows:

(2) $$x^{c}=D_{1}z^{c}\;$$

(3) $$y^{c}=D_{2}z^{c}\;$$

By solving Equations (1)–(3), the $x^{c}$, $y^{c}$, and $z^{c}$ coordinates for each point of the detected object in space are obtained from the absolute phase *φ*.

$\varphi_{0}$ is the absolute phase of the center of the circle at Point B. $\left(u_{0}^{c},v_{0}^{c}\right)$ is principal point of the camera plane. $f_{x}^{c}$ and $f_{y}^{c}$ are the scale factors in the image plane along the $x_{c}$ and $y_{c}$ axes. $\left(u_{0}^{p},v_{0}^{p}\right)$ is principal point of the projector plane. $f^{p}$ is the scale factor in the projector plane along the *x* and *y* axes. R and T are the rotation and translation matrixes from the projector coordinate system to the camera coordinate system. They are expressed as:

$$R=\left[\begin{array}{ccc} r_{11} & r_{12} & r_{13}\\ r_{21} & r_{22} & r_{23}\\ r_{31} & r_{32} & r_{33} \end{array}\right],T=\left[\begin{array}{c} t_{x}\\ t_{y}\\ t_{z} \end{array}\right]\;$$

## 3. Mathematical Model Extension with Least-Squares Parameter Estimation

### 3.1. Error Analysis

**Figure 2:**
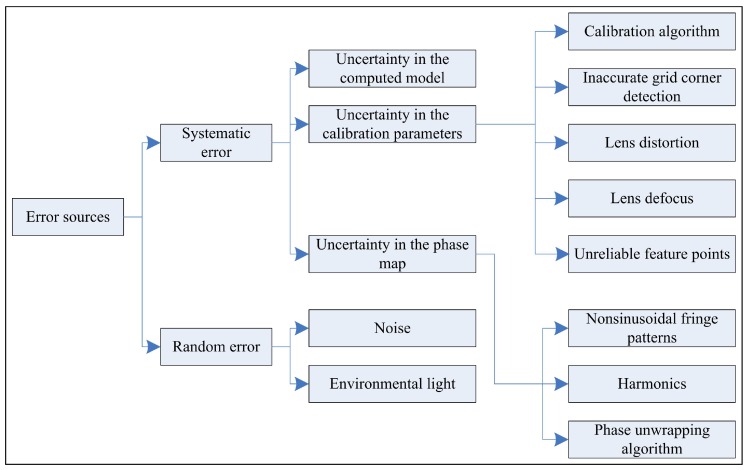
The classification of error sources.

Before working on the mathematical model extension, we first discuss the error sources existing in the fringe projection system. The errors can be considered as a combination of systematic and random errors. A detailed description of the error sources is illustrated in Figure 2.

As for random errors such as noise and the fluctuation in the environmental light, filtering and multiple sampling can be applied to avoid them as much as possible. For systematic errors, from the observation of Equation (1), they include

(1) The uncertainty in the computed model. The simplified geometrical model f(·), which does not consider the lens distortion and lens defocus, results in a systematic error. $\sigma_{f(\cdot )}$ denotes the standard deviation (std) of the simplified model.
(2) The uncertainty in the system calibration parameters. $\sigma_{f_{x}^{c}}$, $\sigma_{f_{y}^{c}}$, $\sigma_{u_{0}^{c}}$, and $\sigma_{v_{0}^{c}}$ denote the std of the estimated camera parameters $f_{x}^{c}$, $f_{y}^{c}$, $u_{0}^{c}$, and $v_{0}^{c}$, respectively; $\sigma_{f^{p}}$ denotes the std of the projector focus length $f^{p}$; $\sigma_{R}$ denotes the std of the rotation matrix between the camera and the projector R; and $\sigma_{T}$ denotes the std of the translation matrix between the camera and the projector T.
(3) The uncertainty in the phase map. The nonsinusoity of the fringe pattern will result in a phase map error. $\sigma_{\varphi }$ denotes the std of the absolute phase map *φ*; $\sigma_{\varphi_{0}}$ denotes the std of the absolute phase of the projection center $\varphi_{0}$.

The whole fringe projection system error $\sigma_{z}$ is accumulated by the abovementioned errors.

### 3.2. Mathematical Model Extension

Considering the complex error sources introduced above, it is difficult to compensate for all of them with a single processing method. In order to eliminate the uncertainty and improve the 3D measurement accuracy with less computational time and complexity, the mathematical model of the phase to 3D coordinates transformation is extended on the basis of the mathematical expression and the uncertainty in the parameters introduced in Section 3.1, and several parameters are added that more accurately describe the relationship between the phase and absolute 3D coordinates. The extended mathematical model is expressed in Equations (4)–(7). $z_{i}^{m}$ is the measured depth value with the extended mathematical expression and expressed as follows:

(4) $$z_{i}^{m}=\frac{K\left(r_{21}^{-1}t_{x}+r_{22}^{-1}t_{y}+r_{23}^{-1}t_{z}+C_{1}\right)-\left(r_{31}^{-1}t_{x}+r_{32}^{-1}t_{y}+r_{33}^{-1}t_{z}+C_{2}\right)}{K\left(r_{21}^{-1}D_{1}+r_{22}^{-1}D_{2}+r_{23}^{-1}+C_{3}\right)-\left(r_{31}^{-1}D_{1}+r_{32}^{-1}D_{2}+r_{33}^{-1}+C_{4}\right)}+K^{\prime }\;$$

where *i* is the ordinal number of each valid point.

(5) $$K=\frac{2\pi (f^{p}+K_{fp})}{\left({\left(\varphi_{u}\right)}_{i}^{m}-\varphi_{0}+K_{\varphi }\right)\lambda_{0}}\;$$

(6) $$D_{1}=\frac{{\left(u_{c}\right)}_{i}^{m}-u_{0}^{c}+K_{u0}}{f_{x}^{c}+K_{fc}}\;$$

(7) $$D_{2}=\frac{{\left(v_{c}\right)}_{i}^{m}-v_{0}^{c}+K_{v0}}{f_{y}^{c}+K_{fc}}\;$$

where $C_{1}$, $C_{2}$, $C_{3}$, $C_{4}$, $K_{fp}$, $K_{\varphi }$, $K_{u0}$, $K_{v0}$, $K_{fc}$, and $K^{\prime }$ are the parameters to be determined. $C_{1}$ and $C_{2}$ are the parameters related to the uncertainty of system calibration parameters R and T. $K_{fp}$ and $K_{fc}$ are the parameters related to the uncertainty of the calibrated focus length of the projector and the camera, respectively. $C_{3}$ and C4 are the parameters related to the uncertainty of rotation matrix R. $K_{\varphi }$ is the parameter related to the phase unwrapping process. $K_{u0}$ and $K_{v0}$ are the parameters related to the uncertainty of the calibrated principle points of the camera. $K^{\prime }$ is the parameter related to the computational model on the phase to depth transformation.

## 4. Realization and Experiments with Single Continuous Objects

### 4.1. Experimental Setup

**Figure 3:**
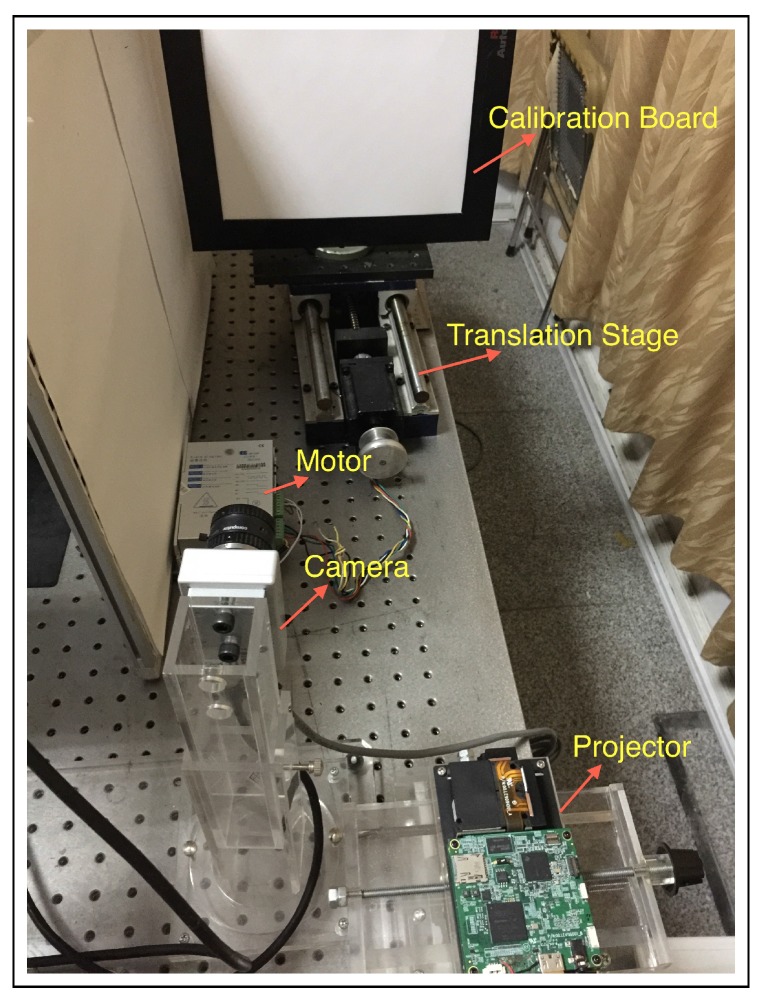
Experimental setup of the fringe projection vision system: Fringe patterns are projected by the projector. The deformed fringe patterns are captured by the camera. The system calibration is implemented with the help of the calibration board.

The experimental setup is shown in Figure 3. The projection unit (DLP LightCrafter, Texas Instruments) projects light patterns onto the target object during measurements. The smart camera (Vision Components VC6210nano, Vision Components GmbH) is responsible for capturing the deformed images. It has an image resolution of 752 × 480 pixels. For the projection unit, the physical resolution of the DLP3000 digital micromirror device (DMD) is 608 × 684 pixels in a diamond array. The DLP3000 DMD is a digitally controlled micro-opto-electromechanical system (MOEMS) spatial light modulator (SLM), which is used to modulate the ampitude and direction of the incoming light. The individual mirror is 7.6 μm square. For a working distance of approximately 600 mm, the width of the projected image is 360 mm; this corresponds to a horizontal resolution of 0.414 mm per pixel assuming “perfect” optics.

### 4.2. System Calibration

**Figure 4:**
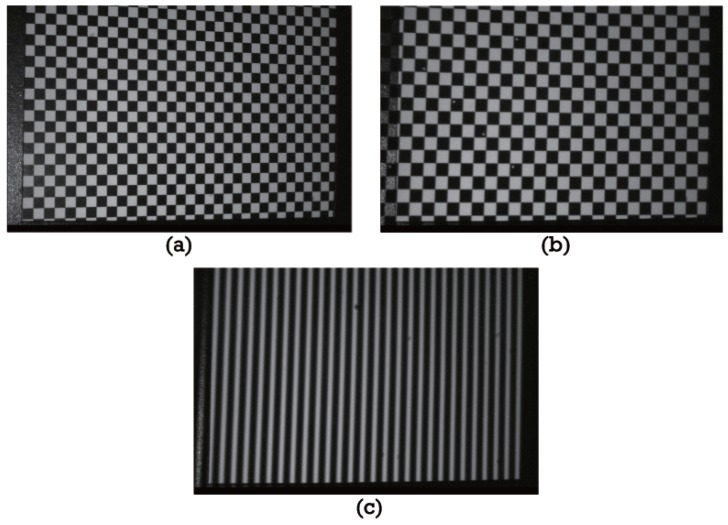
Calibration fringe patterns: (**a**) checkerboard for the camera calibration; (**b**) projected checkerboard for the projector calibration; and (**c**) projected sinusoidal fringe pattern for the depth calculation.

In this study, the plane-based system calibration method is applied to obtain the intrinsic parameters of the camera and projector and their relative positions [23]. Figure 4 shows the calibration patterns. Figure 4a shows the checkerboard pattern for the camera calibration. Figure 4b shows the projected checkerboard pattern for the projector calibration, which covers the same field of view as the camera calibration. In addition, three vertical sinusoidal fringe patterns are projected by the projector and captured by the camera for each calibration position. One example pattern is shown in Figure 4c. The reason for doing this is to obtain the measured depth map for the same calibration position by the three-step phase-shifting technique proposed in this paper.

**Figure 5:**
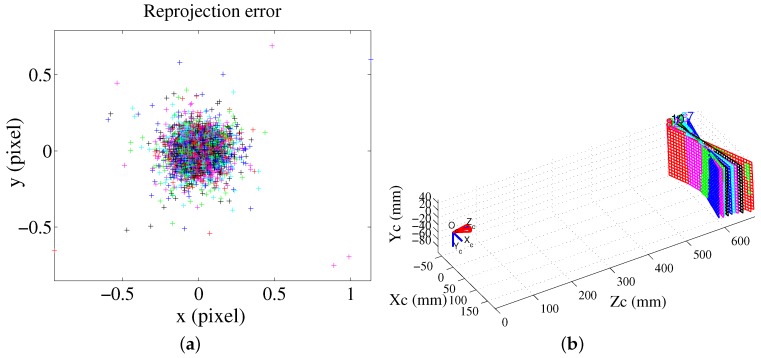
Camera calibration results. (**a**) Reprojection error and (**b**) relative positions of the calibration planes based on the camera coordinate system.

The obtained system calibration is based on the intrinsic parameters of the camera calibration. Therefore, the camera calibration plays an important role in the whole fringe projection 3D measurement. The camera calibration error will be added to the system calibration for the plane-based calibration method. Fortunately, the camera calibration technique is presently very mature and robust. In order to acquire precise camera calibration results, the nonlinear parameter optimization algorithm is adopted by taking full advantage of the geometry of the calibration plane in this paper [24,25,26]. Figure 5 shows the camera calibration reprojection error and the extrinsic parameters for the abovementioned experimental setup. The std of the reprojection error is (0.09870, 0.09053) pixels, and it is equivalent to 0.03 mm for an image resolution of 752 × 480 pixels.

From Figure 5a, the worst reprojection error for some feature points is up to 1 pixel, and the error is up to approximately 0.3 mm, which is not acceptable. For the feature points that are chosen with known geometrical knowledge, we choose points with a reprojection error within 0.25 pixels, which is equivalent to 0.07 mm in the worst case. On the one hand, this still provides enough fitting data to obtain the extended mathematical model parameters. On the other hand, this is the error that we can accept for our fringe projection vision system since the chosen projector of this system has a resolution of 0.414 mm. The std of the reprojection error for the projector is (0.77058, 0.48506), and the reprojection error is equivalent to 0.319 mm, which is much higher than the error deviation of 0.07 mm for the reference coordinates. The 3D information for the calibration plane can be regarded as known geometrical knowledge. The relative positions of the calibration planes based on the camera coordinate system are shown in Figure 5b.

### 4.3. Derivation of the Extended Mathematical Model Parameters

To derive the extended mathematical model parameters introduced in Section 3.2, we first define an error function of the form e(u,v). In the sense of least squares, the parameters can be estimated by minimizing the sum of squares of $e_{i}\left(u_{c},v_{c}\right)$:

(8) $$\min\left\{\sum_{i=1}^{N}e_{i}^{2}\right\}=\min\left\{\sum_{i=1}^{N}{\left(z_{i}^{r}-z_{i}^{m}\right)}^{2}\right\}\;$$

where *i* is the ordinal number of each valid point, and *N* is the total number of valid points that are used in the calculation. $Z_{i}^{r}$ is the reference depth of the $i_{th}$ valid point, which is known in advance as the basic geometric information of the calibration target at different positions from the camera calibration, as introduced in Section 4.2. $Z_{i}^{m}$ is the measured depth of the $i_{th}$ valid point using the extended mathematical model in Equation (4) to the same calibration plane. By solving Equations (4)–(8), the least-squares parameter estimate is implemented. The obtained parameters are as follows:

$C_{1}=0.048$, $C_{2}=0.083$, $C_{3}=0$, $C_{4}=0$, $K_{fp}=0$, $K_{\varphi }=2.7$, $K_{u0}=17.9$, $K_{v0}=0$, $K_{fc}=-6.975$, and $K^{\prime }=0.2$.

### 4.4. Experimental Results

**Figure 6:**
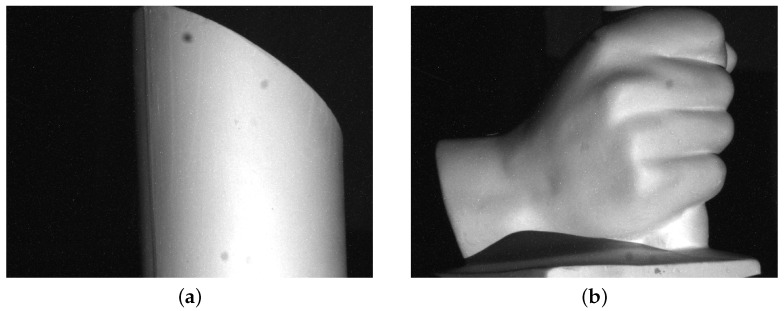
Detected targets. (**a**) Cylinder and (**b**) hand plaster models.

**Figure 7:**
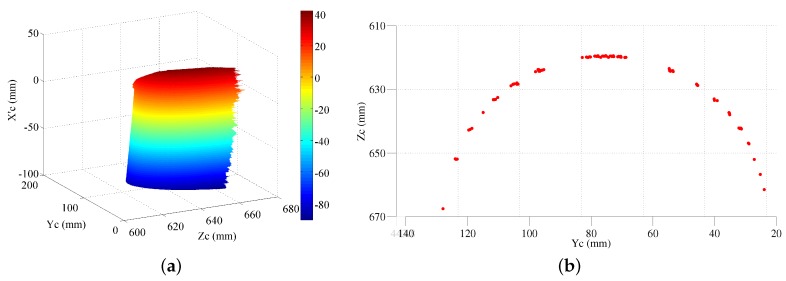
3D measurement by the fringe projection vision system. (**a**) 3D point clouds in Matlab and (**b**) curve of the cross section.

In order to verify the extended mathematical model for the phase to 3D coordinates transformation technique introduced in this paper, we measured the cylinder and hand plaster models shown in Figure 6. Firstly, we carried out the measurement for the cylinder plaster model in Figure 6a with the fringe projection vision system. The point clouds of the measured surface in Matlab are shown in Figure 7a. It should be mentioned that the direction of $X_{c}$ is defined to be inversed to the placement of the detected object. In Figure 7a, $X_{c}^{{}^{\prime }}$ is defined to be opposite to the $X_{c}$ as so to display the measured surface upright. The radius of the cylinder is 52.5 mm. The measured radius is 51.9 mm when Equations (1)–(3) are applied, as shown in Figure 7b. The error is 0.6 mm without using the mathematical model extension technique.

**Figure 8:**
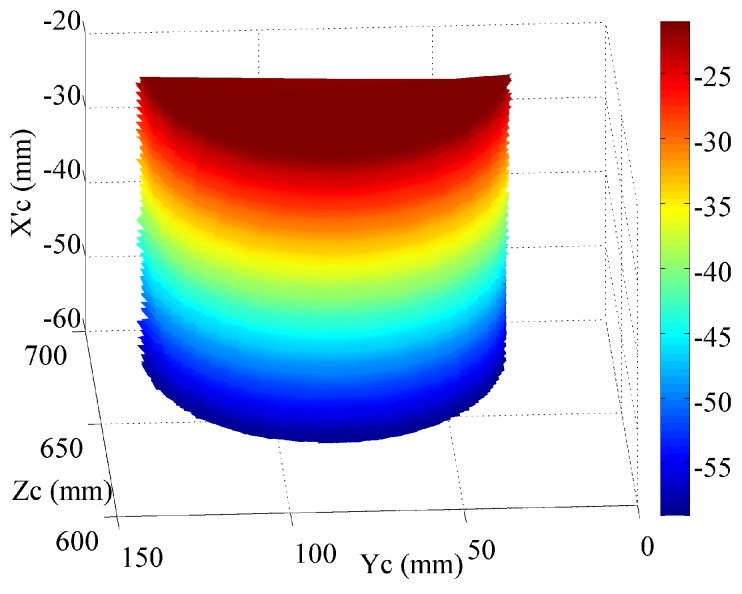
3D measurement by the extended mathematical model.

**Figure 9:**
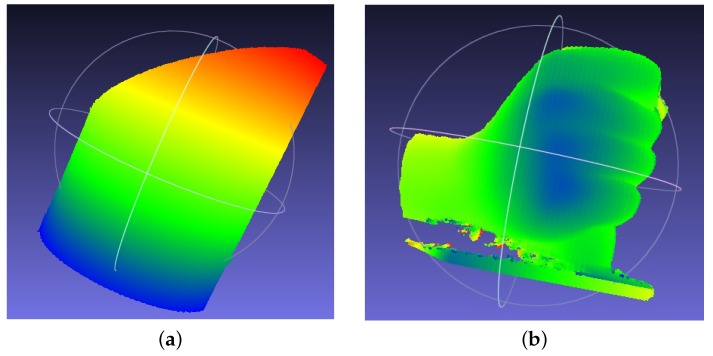
3D measurement by the fringe projection vision system. (**a**) Cylinder and (**b**) hand surfaces displayed with the Meshlab tool.

In order to obtain an accurate 3D measurement, the mathematical model extension technique using least-squares parameter estimation, as introduced in Section 4.3, is applied. After applying Equations (4)–(7) with the abovementioned parameters, the surface of the cylinder is obtained. The terminal points of the cross section to the cylinder surface are A:(52.39,31.27,674.7) and B:(51.78,136.2,680). The connection of Points A and B forms the diameter of the cross section of the cylinder. The calculated radius is 52.53 mm. The error is 0.03 mm when applying the extended mathematical model. In addition, the cylinder with $X_{c}$ from about 20 mm to 60 mm was truncated and shown in Figure 8. A sequence of radius was obtained for the truncated cylinder. The mean value of the measured radius is 52.25 mm. The root mean square deviation (RMSE) of the measured radius is 0.033 mm. It can be clearly seen that the measurement accuracy is significantly improved when using the mathematical model extension technique. The surfaces measured on the cylinder and the hand plaster models with Meshlab are shown in Figure 9. The color changes gradually on the basis of the coordinate $X_{c}$ and $Z_{c}$ for the cylinder and the hand plaster model, respectively.

### 4.5. Performance Comparison with Kinect

**Figure 10:**
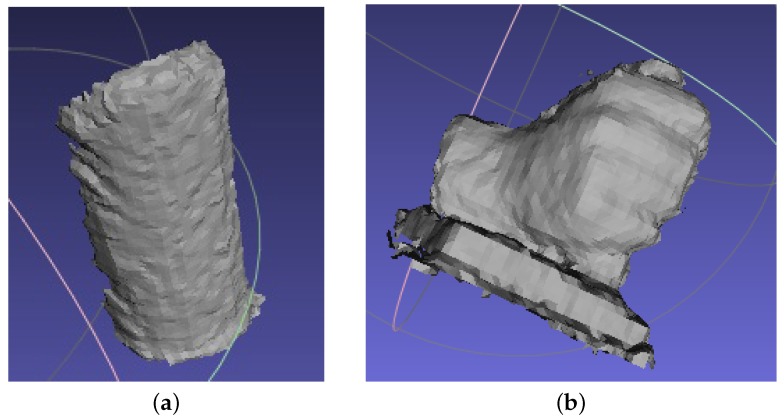
3D measurement with Kinect. (**a**) Cylinder and (**b**) hand surfaces measured with the Meshlab tool.

Similar to the fringe projection vision system described in this paper, Kinect is another kind of structured light technique that uses a speckle pattern to realize a 3D measurement. It is a commercialized product mainly used for gesture control and home entertainment. The depth measurement range for Kinect is 500−3500 mm. It has a resolution of 640 × 480. The fringe projection vision system described in this paper has a working range of 364−2169 mm. Moreover, it has a resolution of 608 × 684. It can be clearly seen that they have very similar measurement specifications. Thus, the detected plaster models are also measured with Kinect in this research work. The detected targets are also placed at a working distance of approximately 650 mm, which is similar to the proposed fringe projection vision system. The measured surfaces are shown in Figure 10.

From Figure 10, we can see that the flatness of the measured surface is much worse compared with the surface measured with the proposed fringe projection vision system in Figure 9. The advantage of Kinect is that it has a special optical design that allows the device to obtain a much larger field of view than the fringe projection vision system proposed in this paper.

## 5. Experiments with Multiple Discontinuous Objects

In phase-shifted fringe projection technique, three sinusoidal fringe patterns are projected onto the object surface with phase shifts of 0, 2π/3, and −2π/3 in this study. The corresponding intensity distributions are as follows:

(9) $$\left\{\begin{array}{c} I_{1}\left(x,y\right)=I^{{}^{\prime }}\left(x,y\right)+I^{\prime \prime }\left(x,y\right)cos\left[\varphi \left(x,y\right)-2\pi /3\right]\\ I_{2}\left(x,y\right)=I^{{}^{\prime }}\left(x,y\right)+I^{\prime \prime }\left(x,y\right)cos\left[\varphi \left(x,y\right)\right]\\ I_{3}\left(x,y\right)=I^{{}^{\prime }}\left(x,y\right)+I^{\prime \prime }\left(x,y\right)cos\left[\varphi \left(x,y\right)+2\pi /3\right] \end{array}\right.\;$$

where $I^{{}^{\prime }}\left(x,y\right)$ is the average intensity, $I^{"}\left(x,y\right)$ is the intensity modulation, and φ(x,y) is the phase to be solved. By solving the above equations, the phase at each point (x,y) of the image plane is obtained as follows:

(10) $$\varphi \left(x,y\right)=\tan^{-1}\left[\frac{\sqrt{3}\left(I_{1}-I_{3}\right)}{2I_{2}-I_{1}-I_{3}}\right]$$

By solving Equation (10), the obtained phase is a relative value between −π and +π. A spatial phase-unwrapping algorithm can be used to remove the 2*π* discontinuities. The obtained absolute phase is

(11) Φ(x,y)=ϕ(x,y)+k(x,y)2π

where k(x,y) is the fringe order.

Nevertheless, for spatially isolated objects, the fringe orders will be ambiguous. It is difficult to retrieve the absolute phase directly. As a result, the depth difference between spatially isolated surfaces is indiscernible. In order to reliably obtain the absolute phase map, a new phase-coding method is proposed that takes full advantage of the frequency characteristic of the sinusoidal fringe pattern.

**Figure 11:**
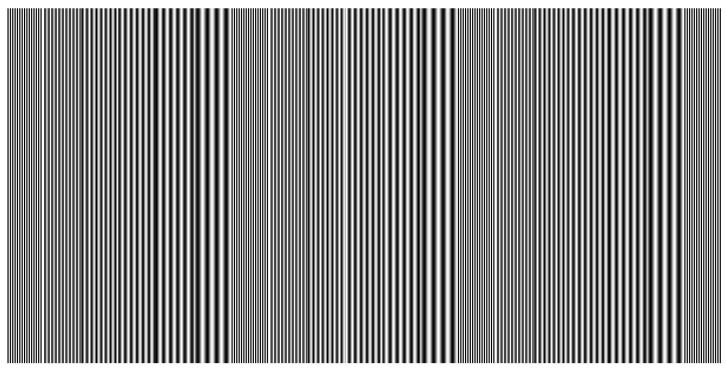
Designed fringe pattern that gives the fringe order information.

**Figure 12:**
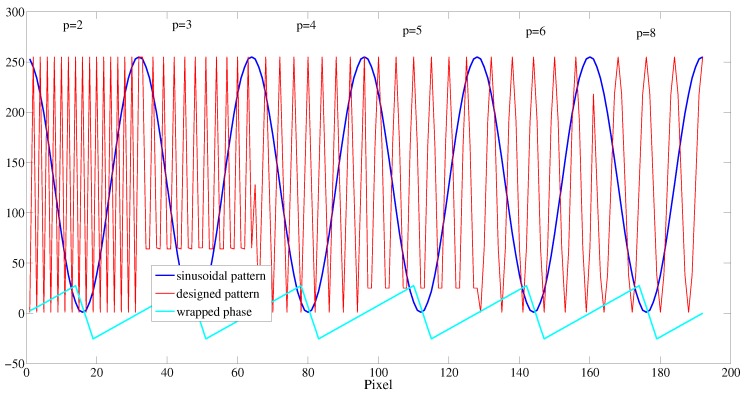
Plot of the sinusoidal fringe pattern with a phase shift of zero that is colored in blue, the phase codeword of the designed fringe pattern that is colored in red, and the wrapped phase that is colored in cyan.

Figure 11 shows the designed fringe pattern. It consists of the codewords that give the fringe order information of *k*. Figure 12 illustrates the relationship among the sinusoidal fringe pattern with a phase shift of zero, the wrapped phase with a relative value between −π and +π, and the codewords of the designed pattern. It should be mentioned that the retrieved wrapped phase is ten times the actual value in order to observe the relationship with the codewords of the designed pattern clearly. The period of the sinusoidal fringe pattern $p_{s}$ is set to 32 pixels in this study. $p_{l}$ is the regional period of the designed codewords. Each codeword consists of a sequence of sinusoidal fringe patterns, and the codewords change from period to period according to the 2π phase-change period. $p_{l}$ equals 2, 3, 4, 5, 6, and 8 pixels separately, as shown in Figure 12. The designed pattern shown in Figure 11 is formed by repeating the codewords $\left(p_{l}=2,3,4,5,6,8\right)$. In addition, when in the system calibration, the projector projects the design pattern to the calibration board, and the camera captures the design pattern. The obtained design pattern is recognized as the template design pattern. It can be used for partition to the detected area to avoid the phase codewords aliasing effect. With the design pattern as shown in Figure 11, there are three partitions at most in the practical application.

From the plot of the codewords of the designed pattern, we know that the frequency component of the codewords changes from high to low. An analysis of the sinusoidal fringe pattern of the designed codewords was carried out for the ideal projected patterns and captured patterns. The results are listed in Table 1. It can be seen that the frequency components for various codewords do not exhibit aliasing. The fringe order *k* in Equation (11) can be reliably obtained on the basis of a frequency analysis of the codewords.

**Figure 13:**
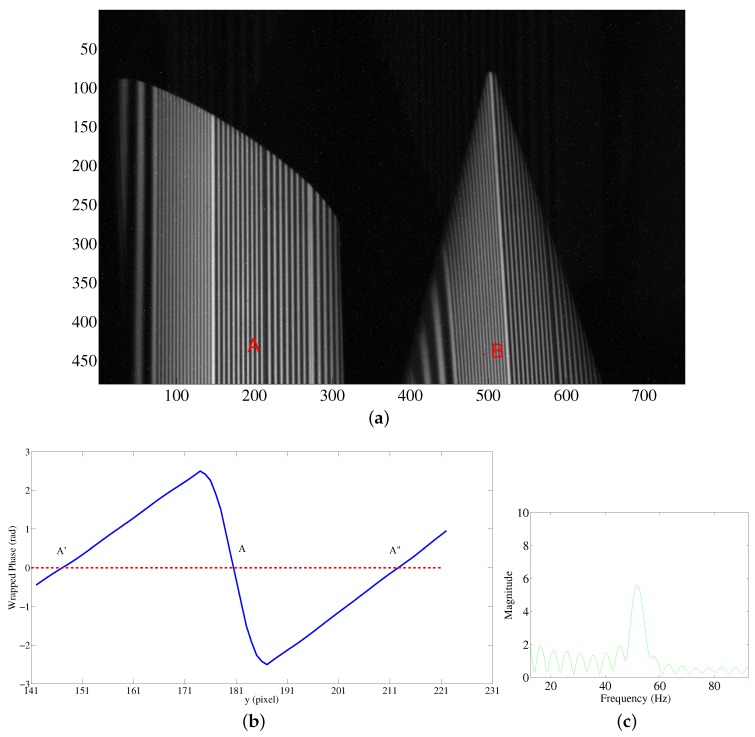
Absolute phase map retrieval for spatially isolated objects. (**a**) Captured designed fringe pattern for multiple discontinuous objects; (**b**) graph for finding the phase zero-crossing points that include the chosen feature point; and (**c**) frequency component obtained by an FFT.

We take one example to describe the framework for obtaining the absolute phase map for multiple discontinuous objects. The detected objects include one cylinder and one cone. They are discontinuous. The designed pattern shown in Figure 11 is projected onto the detected objects. The captured fringe pattern is shown in Figure 13a. The left object is one cylinder. The right object is one cone. The absolute phase map retrieval of the spatially isolated objects is implemented in the following steps:

Step 1: Obtain the wrapped phase from the captured sinusoidal fringe patterns. Segment the whole image into *I* various continuous regions on the basis of the branch-cut map of the wrapped phase. Set the feature point in a continuous region, such as Point A (434,181) and Point B (444,493) in Figure 13a. The chosen feature points are better located in the middle of a 2π phase period as shown in Figure 13a.

Step 2: Adopt the conventional phase-unwrapping algorithm to unwrap the phase for each region to obtain the relative phase map $\Phi_{i}^{r}\left(x,y\right)$ (i=1⋯I). In the example shown in Figure 13a, $\Phi_{cyl}^{r}\left(x,y\right)$ denotes the relative phase map of the cylinder. $\Phi_{con}^{r}\left(x,y\right)$ denotes the relative phase map of the cone. Here we still take the cylinder as the object of research. The absolute phase map of the cone equals its relative phase map as follows:

(12) $$\Phi_{con}^{a}\left(x,y\right)=\Phi_{con}^{r}\left(x,y\right)\;$$

The absolute phase map of the cylinder is obtained as

(13) $$\Phi_{cyl}^{a}\left(x,y\right)=\Phi_{con}^{r}\left(x,y\right)-k*2\pi \;$$

where *k* is the fringe order difference between Points B and A.

Step 3: Obtain the fringe order difference of the phase codeword of Points A and B. We take Point A as an example to explain the retrieval of the fringe order. Firstly, find the 2π phase period that includes Point A and locate the zero-crossing points $A^{\prime }$ and *A*${}^{\prime \prime }$ within the 2π phase period, as shown in Figure 13b. Take a Fast Fourier Transformation (FFT) of this phase period and derive the frequency component, as shown in Figure 13c. The obtained frequency component is about 54 Hz. From Table 1, we know that the phase codeword is p2. Similarly for Point B, the codeword is p1. The difference in the fringe order *k* of Points A and B is 5.

**Figure 14:**
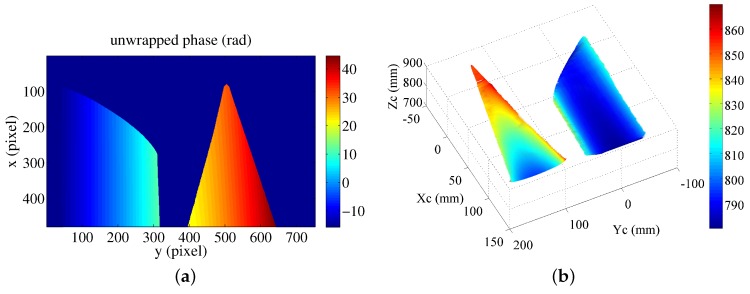

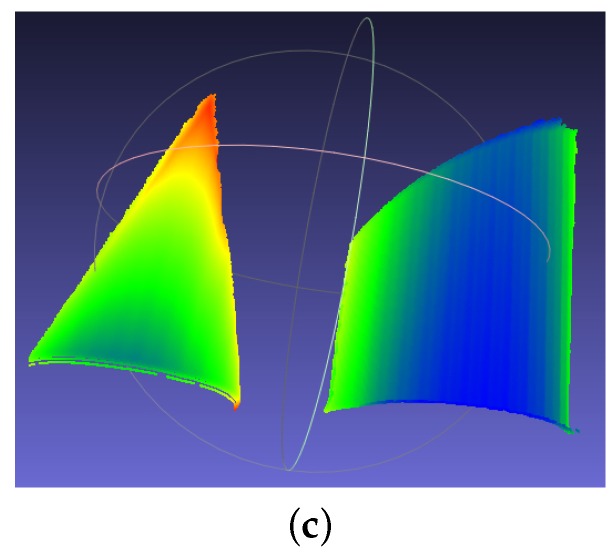
3D measurement by the fringe projection vision system. (**a**) Absolute phase map; (**b**) measured 3D surface for discontinuous objects in Matlab; and (**c**) measured 3D surface for discontinuous objects, shown in Meshlab tool.

On the basis of the Equations (12)–(14), the absolute phase map of the detected objects is derived as shown in Figure 14a. Furthermore, gray-coding plus phase-shifting method is also applied to obtain the absolute phase map of the detected objects. The obtained absolute phase map is the same with the result obtained by the proposed phase-coding method. By applying the abovementioned extended mathematical model, the measured surfaces in Matlab are shown in Figure 14b. The measured surfaces in Meshlab are shown in Figure 14c. The color changes gradually on the basis of the coordinate $Z_{c}$. The calculated radius of the cylinder is 52.56 mm. The error is 0.06 mm. The experimental results demonstrate the effectiveness of the proposed phase-coding method. However, the error is a little larger than the measured radius with single object. More effort will be made to improve the performance of the proposed fringe projection vision system on multiple spatially discontinuous objects.

## 6. Conclusions

In this study, a flexible fringe projection vision system is proposed that relaxes the restriction on the camera’s and projector’s relative alignment and simplifies the calibration process with more flexibility and a cost savings. The proposed flexible fringe projection vision system is designed to realize an accurate 3D measurement to large scene for industrial safety inspection. The measured range can be flexibly adjusted on the basis of the measurement requirements. Accurate 3D measurement is obtained with an extended mathematical model that avoids the complex and laborious error compensation procedures for various error sources.

In addition, the system accuracy is only determined by the derived point clouds with a mature camera calibration technique. It is not influenced by the numerous systematic error sources. Moreover, the system accuracy can be further improved by the following ways. (1) In this paper, a low-cost printed plane is used for the calibration; if a high-accuracy planar plane is used, the calibration accuracy can be further improved, and the known geometrical information (the calibration reference plane) will provide a more accurate reference value for the following nonlinear parameter estimation; (2) A high-resolution camera is applied. For the vision system in this work, the std of the reprojection error is 0.03 mm, and it can be reduced to 0.015 mm with a camera with a two-fold higher resolution; (3) A more advanced camera calibration algorithm with highly accurate feature point extraction can be used.

Finally, a new phase-coding method is proposed to obtain the absolute phase map of spatially isolated objects. Only one additional fringe pattern is added for the proposed phase codeword method. There is no gray level or color information that is used to unwrap the phase with depth discontinuities because they are sensitive to noise. Since the proposed method takes full advantage of the frequency characteristic of the sinusoidal fringe pattern, it is insensitive to noise and object texture. However, accurate retrieval of the fringe order is dependent on the FFT of the signal of the phase period. If the signal is too short, the accuracy of the fringe order retrieval will be affected. In our future work, it will be improved by combining the FFT and a correlation technique for the phase period signal.

## Figures and Tables

**Table 1:** Frequency component of each phase codeword.

Codeword	Period	Ideal Sinusoidal Fringe Pattern	Captured Sinusoidal Fringe Pattern
p1	2	80.5	82 ± 1
p2	3	53.2	54 ± 0.5
p3	4	40.5	41 ± 0.5
p4	5	32.6	33.5 ± 0.5
p5	6	27.3	28 ± 0.5
p6	8	20.5	21 ± 0.5
